# Prognostic Factors for Checkpoint Inhibitor Based Immunotherapy: An Update With New Evidences

**DOI:** 10.3389/fphar.2018.01050

**Published:** 2018-09-20

**Authors:** Xinyu Yan, Shouyue Zhang, Yun Deng, Peiqi Wang, Qianqian Hou, Heng Xu

**Affiliations:** ^1^Department of Laboratory Medicine, Research Center of Clinical Laboratory Medicine, West China Hospital, Sichuan University, Chengdu, China; ^2^State Key Laboratory of Oral Diseases, West China Hospital of Stomatology, Sichuan University, Chengdu, China; ^3^State Key Laboratory of Biotherapy, West China Hospital, Sichuan University and Collaborative Innovation Center, Chengdu, China; ^4^Precision Medicine Center, State Key Laboratory of Biotherapy and Precision Medicine, Key Laboratory of Sichuan Province, West China Hospital, Sichuan University and Collaborative Innovation Center, Chengdu, China

**Keywords:** immunotherapy, checkpoint inhibitor, PD-1, PD-L1, CTLA-4

## Abstract

Checkpoint inhibitor (CPI) based immunotherapy (i.e., anit-CTLA-4/PD-1/PD-L1 antibodies) can effectively prolong overall survival of patients across several cancer types at the advanced stage. However, only part of patients experience objective responses from such treatments, illustrating large individual differences in terms of both efficacy and adverse drug reactions. Through the observation on a series of CPI based clinical trials in independent patient cohorts, associations of multiple clinical and molecular characteristics with CPI response rate have been determined, including microenvironment, genomic alterations of the cancer cells, and even gut microbiota. A broad interest has been drawn to the question whether and how these prognostic factors can be used as biomarkers for optimal usage of CPIs in precision immunotherapy. Therefore, we reviewed the candidate prognostic factors identified by multiple trials and the experimental investigations, especially those reported in the recent 2 years, and described the possibilities and problems of them in routine clinical usage of cancer treatment as biomarkers.

## Introduction

Existence of immune checkpoints is essential for modulating duration and magnitude of T cell responses and maintaining self-tolerance (Pardoll, 2012), while suppression of antitumor immune responses facilitates harmful tumor growth. With a constantly deepening understanding of the immune system and its role on cancer development, the field of cancer immunotherapy has been explored with great enthusiasm, aimed at harnessing immune system to induce or restore antitumor activities (Topalian et al., 2011). Among complicated pathways of immune system, interactions of cytotoxic-T-lymphocyte-associated protein 4 (CTLA-4) with CD80/CD86, and programmed cell death 1 (PD-1) with programmed cell death ligand 1 (PD-L1) has been considered to act as “brakes” on the immune system (Linsley et al., 1991; Freeman et al., 2000; Schildberg et al., 2016). CTLA-4 has a much stronger affinity with CD80/86 than CD28, thus inhibiting crucial CD28/CD80 and CD28/CD86 based T cell activation (Manson et al., 2016), while PD-1/PD-L1 interaction induces imbalanced activation of signaling pathways which results in altered T-cell metabolism and subsequent abnormal differentiation, leading to reduced T effector cells and increased T regulatory cells (Tregs) as well as T exhausted cells (Boussiotis, 2016). Therefore, CTLA-4 and PD-1/PD-L1 have been considered as the “star” candidate targets to immune-checkpoint blockade (ICB) based immunotherapy. Unprecedented success of anti-CTLA-4 and anti-PD-1/PD-L1 ICBs have been achieved in various tumor types that were previously sentenced to gloomy prognosis under traditional treatments (Thomas and Hassan, 2012; Gogas et al., 2013; Lee et al., 2015; Restifo et al., 2016), significantly prolonging overall survival with acceptable toxicity in patients with advanced melanoma (Hodi et al., 2010; Wolchok et al., 2013; D’Angelo et al., 2017), non-small-cell lung cancer (NSCLC) (Gettinger et al., 2015, 2016; Hellmann et al., 2017), and other tumor types (Hamanishi et al., 2015; Morris et al., 2017; Overman et al., 2017).

Until recently, six CPIs have been approved by the U.S. Food and Drug Administration (FDA), and all of them are monoclonal antibodies against the targets, including one for CTLA-4 (i.e., Ipilimumab), two for PD-1 (i.e., Pembrolizumab and Nivolumab), and three for PD-L1 (i.e., Avelumab, Atezolizumab, and Durvalumab) (**Table 1**). Ipilimumab was firstly approved for advanced melanoma in 2011 (Ma et al., 2016), which symbolizes the remarkable clinical success of anti-CTLA-4 and thus elicits further investigations into PD-1/PD-L1 pathway. Pembrolizumab was the first inhibitor for PD-1, which was approved as the second-line treatment for unresectable or metastatic melanoma, followed by Nivolumab (for unresectable metastatic melanoma, advanced metastatic NSCLC and advanced metastatic renal cell carcinoma), Atezolizumab (for urothelial carcinoma following platinum-based chemotherapy), Avelumab (for metastatic Merkel-cell carcinoma, and Durvalumab for urothelial carcinoma following platinum-based chemotherapy) (Manson et al., 2016; Pitt et al., 2016). Afterward, indications of these CPIs have been largely expanded after clinical trials, and exhibits remarkable disease responses in a wide range of histological types of carcinomas, such as hematologic malignancies, head and neck cancer, and bladder cancer (Armand et al., 2013; Postow et al., 2015a; **Table 1**). Recently, Nivolumab has been successfully used as a neoadjuvant therapy before surgery in patients with early untreated NSCLC, and preoperative usage of Nivolumab can induce augmentation of neoantigen-specific T cells (Forde et al., 2018). Noteworthily, though sharing almost similar mechanisms, anti-PD-L1 therapy may render distinct effect from anti-PD-1. The subtle difference lies in that the PD-L1 antibody does not block the interaction between PD-1 and PD-L2, while PD-1 blockade cannot block the interaction of PD-L1 with CD80, which is expressed on T cells and deliver inhibitory signals of antitumor activities (Butte et al., 2007). Actually, a meta-analysis has shown that anti-PD-1 achieves higher overall survival and response rate than anti-PD-L1 in NSCLC, which reveals anti-PD-1 as a better choice for patients with NSCLC (You et al., 2018). Moreover, accumulated evidence has indicated that combined usage of anti-PD and anti-CTLA-4 antibodies can synergetically improve clinical outcome compared with either agent alone (Larkin et al., 2015; Hodi et al., 2016; Hellmann et al., 2017; Wolchok et al., 2017), probably due to their different function mechanisms.

**Table 1:** FDA-approved immune checkpoint inhibitors in cancer treatment.

Target	Antibody	Trade name	Company	Indication (approval date)
CTLA-4	Ipilimumab	YERVOY	Bristol-Myers Squibb (BMS)	Unresectable or metastatic melanoma (2011)
PD-1	Pembrolizumab	KEYTRUDA	Merck Sharp & Dohme (MSD)	Unresectable or metastatic melanoma (approved for patients with disease progression after ipilimumab and, if BRAF V600 mutation positive in 2014, and expanded to initial treatment in 2015)
				Metastatic NSCLC whose tumors express PD-L1 as determined by an FDA-approved test and who have disease progression on or after platinum-containing chemotherapy (2015)
	Nivolumab	OPDIVO	Bristol-Myers Squibb (BMS)	Metastatic melanoma (2014, approved for BRAF V600 wild-type tumor in 2015)
				Squamous NSCLC with progression or after platinum-based drugs (2015, and expanded to non-squamous NSCLC later in 2015)
				Advanced metastatic renal cell carcinoma after angiogenic therapy (2015)
				Classical Hodgkin lymphoma that has relapsed or progressed after autologous hematopoietic stem cell transplantation and post-transplantation brentuximab vedotin (2016)
				Locally advanced or metastatic urothelial carcinoma which have progression during or following platinum-containing chemotherapy or have progression within 12 months of neoadjuvant or adjuvant treatment with platinum-containing chemotherapy (2017)
PD-L1	Atezolizumab	TECENTRIQ	Roche and Genentech	Locally advanced or metastatic urothelial carcinoma after failure of cisplatin-based chemotherapy (2016), but the confirmatory trial failed
				Metastatic NSCLC whose disease progressed during or following platinum-containing chemotherapy (2016)
	Avelumab	BAVENCIO	Merck and Pfizer	Metastatic Merkel-cell carcinoma (2017)


Although great success has been achieved with CPI based immunotherapy, large individual differences were noticed in terms of treatment outcomes (Gibney et al., 2016; Manson et al., 2016; Pitt et al., 2016; Topalian et al., 2016; Zou et al., 2016; Nishino et al., 2017), which varied among different cancer types. For instance, the response rate for patients treated with Ipilimumab is only 10–15% in metastatic melanoma (Hodi et al., 2010), and rarely exceeds 40% for PD-1 blockade therapy, even a large proportion of partial responders were included (Brahmer et al., 2012; Hamid et al., 2013), indicating that the majority of patients treated with PD-1/PD-L1 blockade fail to respond sufficiently. In addition, PD-1/PD-L1 blockade can induce immune-related adverse drug reaction events (ADR) deriving from non-specific immunologic activation, which are reported to be much less than those induced by anti-CTLA-4, though (Larkin et al., 2015; Robert et al., 2015). The toxicities observed in CPI treatment include the most frequent fatigue and possibly fatal inflammatory pneumonitis, and high grade adverse events may lead to forced abortion of the treatment (Zou et al., 2016). Worse still, some patients even demonstrate disease hyperprogression following treatment, which is defined as <2 months of time-to-treatment failure (TTF), >50% increase in tumor burden compared with preimmunotherapy imaging, and >2-fold increase in progression pace (Champiat et al., 2017; Kato et al., 2017). In this case, effective biomarkers for the indication of treatment outcomes are largely required. Indeed, some biomarker candidates have been put into practice, and recommended to be determined before CPI treatments.

**FIGURE 1:**
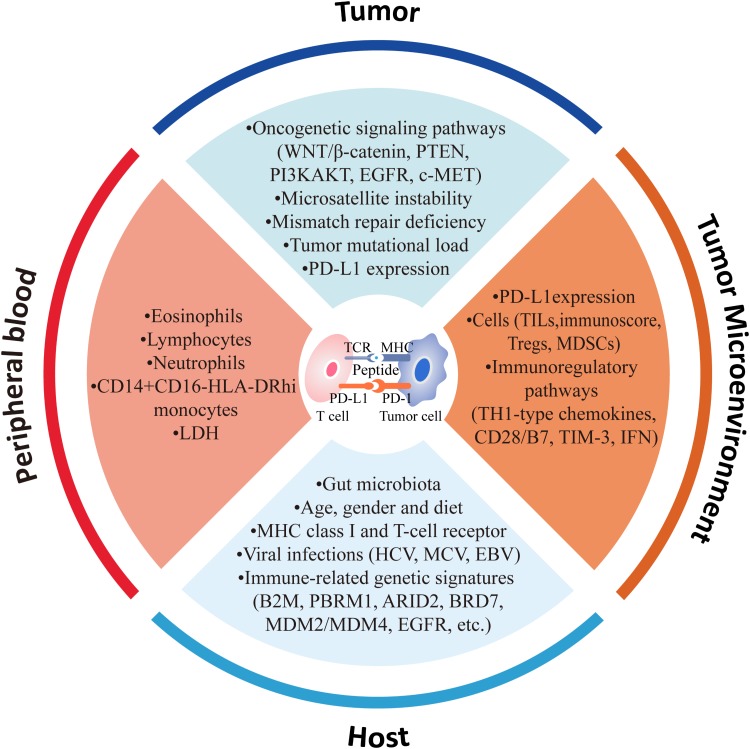
Factors associated with response to anti-PD-1/PD-L1 therapy.

In precision medicine era, understanding the mechanisms, by which patients lack response/produce resistance to CPI treatments or suffer from severe ADR, is of utmost importance for selecting the patients specifically suitable for the treatment. In this review, we will focus on current knowledge of factors that influence the sensitivity and resistance to CPI-based immunotherapy (e.g., clinical characteristics, genomic alterations, tumor microenvironment (TME), host immune functions, and gut microbiota), and highlight the potential biomarkers for CPI treatments, especially the new evidences reported lately (**Table 2** and **Figure 1**).

**Table 2:** Factors related to the efficacy of ICBs.

Classification	Biomarkers	Influence
Clinical-relevant factors	Age	The elderly patients lack response to ICBs.
	Gender	Male patients respond better to ICBs.
	Diet	Obesity and improved FA catabolism improve anti-PD therapy.
	Viral infection	MCV and EBV infected patients respond better to anti-PD therapy.
Tumor autonomous mechanisms	Tumor mutational/neoantigen load	High mutational/neoantigen loads improve efficacy of ICBs
	PD-L1 expression	High PD-L1 expression improves anti-PD therapy
Tumor microenvironment	Cells	Increased TILs improve response to ICBs, while Tregs and MDSCs impair the efficacy.
	Immunoregulatory pathways	Inhibition of TH1 chemokines, CD28/B7, IFN and activation of TGFβ, TIM3 lead to resistance to PD blockades.
Host-related factors	Peripheral blood markers	Increased eosinophils, lymphocytes, monocytes and low LDH levels improve response to PD blockades.
	MHC class I	Impaired MHC class I molecules lead to resistance to anti-PD therapy
	TCR repertoire	Less diverse T cell repertoire improves response to anti-PD
	The gut microbiota	*Bacteroides* species facilitate anti-CTLA, more diversified bacteria, such as *Bifidobacterium, Akkermansia muciniphila, Ruminococcaceae bacteria*, facilitate anti-PD.


## Clinically Relevant Factors

### Age, Gender, and Diet

Aging is commonly correlated with limited and dysfunctional immune activities characterized by reduced lymphocyte proliferation and increased exhausted T cells, resulting in susceptibility to various diseases and increased cancer incidence (Fulop et al., 2010; Lee et al., 2016). *In vivo* studies have shown upregulation of PD-1 expression on T cells of aged animals, indicating the potentially critical role of PD-1 blockades in the old (Mirza et al., 2010; Lim et al., 2015). Consistent with the decreased activity of immune system in elders, current evidence exhibited that ICB therapy can significantly benefit all age of patients with NSCLC with the exception of patients ≥75 years (Landre et al., 2016; Nishijima et al., 2016; Ferrara et al., 2017). In another hand, anti-PD-1/PD-L1 is found to be capable of inducing hyperprogressive disease during the treatment, which is more frequent in elderly patients (Champiat et al., 2017). Therefore, the age at diagnosis may influence the efficacy and side ADR rate of CPI treatments, although more confirmation investigations with larger samples and less heterogeneity are warranted to settle this debated topic.

Substantial sex-dependent diversities in innate and adaptive immunity have been noticed for a long time, resulting in different susceptibility and immune functions in response to infections and autoimmune diseases between males and females (Fischer et al., 2015; Klein and Flanagan, 2016). Interestingly, accumulated evidence has highlighted that gender plays a considerable role in response to CPIs. A systematic review on the relationship between efficacy and sex of patients indicates that the efficacy of CPI based treatments is sex-dependent, with significantly greater benefit in male patients in all studied cancer types (Conforti et al., 2018). Likewise, another study shows that more improvement of survival resulting from CPI treatment is observed in males than females, and the survival of patients treated with anti-CTLA-4 is more influenced by sex compared with those receiving anti-PD-1 (Wu et al., 2018). Though the current conclusions are not confirmed and clinical trials including more female patients are needed, the gender of patients should be taken into consideration in CPI based treatments.

Healthy diet including sufficient nutrient intake is of great significance for maintaining powerful immune defense against invading pathogens, especially for patients combating tumor progression. It is well reported that unbalanced diet may lead to impaired immunity and accelerate disease development, and obesity is associated with chronic inflammation and cancer development (Fang et al., 2017; Quail et al., 2017). Paradoxically, a meta-analysis of patients with metastatic melanoma indicates that obesity is correlated with improved benefit of anti-PD therapy compared with normal body-mass index (BMI) (McQuade et al., 2018). Interestingly, this association is only observed in males without any clear mechanisms clarified. Moreover, dysregulated metabolism may contribute to the exhaustion of lymphocyte infiltration within the TME. For example, it has been recently discovered that CD8 + T cells enhance peroxisome proliferator-activated receptor (PPAR)-α signaling and catabolism of fatty acids when simultaneously subjected to hypoglycemia and hypoxia. Promoting fatty acid catabolism obviously improves the capacity of tumor infiltrating lymphocytes (TILs) to delay tumor growth and synergizes with PD-1 blockade to efficiently boost the efficacy of melanoma immunotherapy (Zhang Y. et al., 2017). Through influencing multiple immune components and functions, diet and metabolic factors might be related to clinical effect of PD-1 blockade, though direct evidence is currently lacked.

### Viral Infections

Disorders of the immune system and failure in tumor eradication can result from viral infections, which may also impact the ICB treatment response. For instance, a clinical observation regarding advanced Merkel-cell carcinoma exerts significantly high level of clinical response, providing a novel perspective that virus-positive status may contribute to success of anti-PD-1 therapy (Nghiem et al., 2016). Theoretically, oncogenic viruses may serve as strong tumor-specific antigens, and cancer cells should escape from the immune monitoring through inducing immune inhibition. In fact, overexpression of PD-L1 is commonly observed in Merkel-cell carcinoma cells (Wong et al., 2015). Similarly, Epstein-Barr virus (EBV)-positive gastric cancer has been recently reported to have low mutation burden but high expression of immune checkpoint pathways and abundant lymphocytic infiltration, thus demonstrating meaningful clinical response to PD-1/PD-L1 inhibitors (Janjigian et al., 2017; Panda et al., 2017). It has been further discovered that part of CD8 + TILs can recognize tumor unrelated epitopes, such as those from EBV, human cytomegalovirus and influenza virus, which may explain the mechanism by which virus-positivity facilitates host immunity. Moreover, these CD8 + TILs lack the expression of CD39, suggesting that measuring CD39 expression could be an effective approach to select the patients with high possibility of virus infection (Simoni et al., 2018). Although more virus related ICB treatment trials with larger sample size are warranted, current evidence implies oncogenic viruses may be considered as a potential biomarker for predicting effect of anti-PD therapies.

## Tumor Autonomous Mechanisms

### Tumor Mutational Loads, Mismatch Repair Deficiency, and Microsatellite Instability

Tumor mutational burden (TMB), which is mostly determined by next generation sequencing, has been broadly found to be associated with the response to CPIs. Evidence from clinical trials suggests the positive correlation between high tumor mutational loads and improved clinical efficacy of ICB-based therapies (including anti-PD-1, anti-PD-L1, and anti-CTLA-4) in NSCLC and melanoma (Snyder et al., 2014; Rizvi et al., 2015; Van Allen et al., 2015; Hugo et al., 2016; Forde et al., 2018), which have the highest mutation burdens as well as response rates (Lee et al., 2010; Berger et al., 2012; Topalian et al., 2012). Actually, a pooled analysis across 27 tumor types or subtypes illustrated a significantly strong positive correlation between the TMB and the objective response rate to PD-1 inhibition (Yarchoan et al., 2017), indicating the biomarker potential of TMB for PD-1 blockade efficacy. Besides, TMB also predicts clinical efficacy in the combination of anti-PD-1 and anti-CTLA-4 (Hellmann et al., 2018). Loss-of-function of alterations in genes involved in DNA repair can largely induce high TMB, and lack of the ability to repair DNA errors is closely related to microsatellite instability (MSI). Therefore, remarkable clinical benefit from ICB therapy are significantly enriched in patients with MSI status (Le et al., 2015) or specific alterations in DNA repair genes, such as *BRCA2*, *POLD1, POLE*, and *MSH2* (Rizvi et al., 2015; Hugo et al., 2016). Due to the stronger practicality, clinical examination of MSI status, deficiency of mismatch repair genes (through immunohistochemistry), or Lynch Syndrome (inherited mutations in mismatch repair genes with family history) can efficiently predict the good responders, although some patients with negative signals of these potential biomarkers may still get benefit from ICB treatments (Dudley et al., 2016).

It is considered that better response of patients with high TMB to ICB response is attributed to immunogenicity of tumor cells, somatic mutations of which can be translated to antigens and recognized as tags of “foreign” by the immune system (Gibney et al., 2016). These tumor-specific antigens are named as “neoantigens,” and thereby provide highly specific targets for anti-tumor activities of the immune system (Hacohen et al., 2013; van Rooij et al., 2013). The process of neoantigen recognition is attenuated by expression of PD-L1 and some other immunosuppressive ligands (Pages et al., 2005; Llosa et al., 2015). Hence, blockade of immune checkpoints will release inhibition of immune system and reinvigorate pre-existing neoantigen recognition. Not surprisingly, neoantigen burden is closely correlated to TMB, and can be also induced by mismatch repair deficiency (Le et al., 2015). Quite a few patients with advanced mismatch repair-deficient cancers demonstrate significantly durable responses to PD-1 blockade with expanded neoantigen-specific T cell clones (Le et al., 2017). Additionally, neoantigens are mostly predicted by bioinformatic approaches with computational algorithms, which is highly imperfect in terms of low validation rate (e.g., 1–3 mutation-associated neoantigens out of top 30–50 predicted candidates validated by T cell responses) (Kvistborg et al., 2014; Tran et al., 2015), while it is complicated and time-consuming to determinate the functional neoantigens with a series of immunologic experimental investigations, making it improper for neoantigens as an effective clinical biomarkers so far.

Few but important exceptions rejecting the predictive role of tumor mutational status exist in the aforementioned studies (Rizvi et al., 2015; Hugo et al., 2016), consistent with a finding that tumor infiltration is not weakened under the circumstance of low mutational loads in gastrointestinal cancers (Tran et al., 2015), indicating other equally considerable mechanisms contributing to treatment resistance. Neoantigen intratumour heterogeneity may play an important role, and patients with both high TMB and low neoantigen intratumour heterogeneity (<1%) have significantly longer progress-free survival and overall survival compared to patients with high TMB alone (McGranahan et al., 2016). Moreover, strong antigens may disobey the correlation of neoantigen and TMB. For instance, Merkel cell polyomavirus (MCV)-associated Merkel-cell carcinomas have a 100 times lower mutational load than ultraviolet-induced virus-negative Merkel-cell carcinomas (Wong et al., 2015; Goh et al., 2016), but exhibit better response to ICB therapy, which can be explained by its presentation of strong viral antigens (Yarchoan et al., 2017).

### PD-L1 Expression

Increased PD-1 ligands and their ligation to PD-1 on tumor-specific CD8 + T cells is a pivotal strategy adopted by tumors to contend with host immune responses. In certain cancer types (e.g., melanoma, NSCLC, pancreatic cancer, breast cancer, and gastrointestinal stromal tumors), PD-L1 expression is upregulated and associated with poor prognosis (Konishi et al., 2004; Bertucci et al., 2015; Sabatier et al., 2015; Birnbaum et al., 2016). Tumor PD-L1 upregulation reflects negative dynamic immune activities in the TME (Taube et al., 2012; Spranger et al., 2013) and is the premise of anti-PD-1/PD-L1 therapy. So far, PD-L1 is one of the best-studied as well as widely used biomarkers.

Studies on NSCLC have shown that patients with high expression of PD-L1 on the surface of tumor cells have significantly better clinical responses to PD-1/PD-L1 inhibitors (Passiglia et al., 2016; Muller et al., 2017). Likewise, patients treated with the anti-PD-1 antibody BMS-936558 (also known as MDX-1106) respond differently according to their PD-L1 status (Brahmer et al., 2010; Topalian et al., 2012). In a meta-analysis of patients treated with Nivolumab, Pembrolizumab or MPDL3280A (an engineered anti-PD-L1 antibody), response rates are significantly higher in PD-L1-positive tumors, and the predictive role of PD-L1 on tumor cells is stronger for Pembrolizumab and Nivolumab (Carbognin et al., 2015). Samples from several cancer types demonstrate that response to anti-PD-1 blockade is most closely correlated with the expression of tumor cell PD-L1 in comparison with that of other immunosuppressive molecules such as PD-1 and PD-L2 (Taube et al., 2014). On the other hand, in addition to PD-L1 expressed on tumor cells, PD-L1 expression on tumor infiltrating cells also displays noteworthy connections with clinical outcome of MPDL3280A (Herbst et al., 2014; Powles et al., 2014).

PD-L1 immunohistochemistry (IHC) has been approved by FDA as a companion diagnostic to select patients with NSCLC suitable for Pembrolizumab treatment. Nevertheless, absence of PD-L1 does not necessarily imply poor response to anti-PD-1/PD-L1 blockades. Some patients with low PD-L1 expression still demonstrate impressive clinical effect. The paradoxical predictive value of PD-L1 expression may partly be explained by different standards of analyzing, including different staining techniques or assessed range (tumor or both tumor and cells in microenvironment). The different threshold of PD-L1 expression is also important. A good example is the clinical trials of Nivolumab vs. Pembrolizumab as first-line treatment. Nivolumab was the firstly emerged anti-PD-1 CPI, however, failed in clinical trials probably because of the low setting of PD-L1 expression threshold at >1%. On the contrary, Pembrolizumab was later developed and precisely applied to the patients with PD-L1 expression >50% in clinical trials, which made it successfully become the first-line treatment for NSCLC. Besides, dynamic and inducible characteristic of PD-L1 expression also contributes to the contradictory results. PD-L1 can be up-regulated by IFNγ, hence patients with low baseline PD-L1 level may gradually become strong PD-L1 positive under an inflammatory circumstance as the treatment proceeds, and the response to anti-PD blockade also changes as PD-L1 is upregulated (Manson et al., 2016; Zou et al., 2016). Therefore, the application of PD-L1 expression assessment is endowed with useful but not definitive predictive value.

In another hand, further efforts are still needed to refine the clinical use of PD-L1 expression as biomarkers, especially detected by immunohistochemistry. Firstly, PD-L1 expression may be checked in multiple sites of tumor at multiple time points, because PD-L1 expresses dynamically and thus can be influenced by different mechanisms; secondly, standardized determination of PD-L1 expression is largely needed to exclude the possible variation induced by different PD-L1 antibodies (Gibney et al., 2016).

### Gene Mutations and Genomic Alterations in Tumor

Cancer cell genetic alterations in pivotal signaling pathways might be responsible for suppressed T cell activities and deficient antitumor immunity, consequently impacting response to anti-PD therapies (**Table 3**). Tumor-intrinsic activation of WNT/β-catenin signaling pathway results in subdued CCL4 expression and subsequent precluded dendritic cell (DC) recruitment and DC-mediated T-cell activities, thus leading to resistance to anti-PD-L1 and anti-CTLA-4 therapies (Spranger et al., 2015). Loss of phosphatase and tensin homolog (PTEN) as well as activation of PI3K-AKT pathway in tumor cells brings about increased immunosuppressive cytokines and attenuated T-cell infiltration and activity, thereby promoting resistance to PD-1 inhibitor therapy (Peng et al., 2016). Similarly, EGFR pathway activation has been found to be correlated with development of immunosuppressive microenvironment represented by upregulation of PD-1, PD-L1, CTLA-4, and multiple tumor-promoting inflammatory cytokines (Akbay et al., 2013). Patients with *EGFR* mutation even receive less benefit from ICB therapy compared to chemotherapy (Borghaei et al., 2015; Rittmeyer et al., 2017). Clinical data of patients with NSCLC shows that mutations in *EGFR* are associated with low overall response rate to PD-1/PD-L1 inhibitors due to decreased PD-L1 expression and CD8 + TILs. However, T790M-negative *EGFR*-mutant patients are more likely to benefit from anti-PD-L1 after previous treatment (Gainor et al., 2016; Haratani et al., 2017). In addition to poor outcome, patients with *EGFR* alterations tend to be hyperprogressors with significantly increased tumor growth rate after receiving PD-1/PD-L1 inhibitors (Kato et al., 2017). In the other hand, recent evidence indicates that inhibitors of the receptor tyrosine kinase c-MET impair reactive mobilization and recruitment of neutrophils into tumors and draining lymph nodes, and thus increase effector T cell infiltration, suggesting c-MET pathway inhibition may improve responses to checkpoint immunotherapies including anti-PD (Glodde et al., 2017).

**Table 3:** Alterations of genes associated with effect of anti-PD therapy.

Gene	Change of the response caused by mutations	Mechanism
*BRCA2*	Better	Mismatch repair deficiency (Hugo et al., 2016)
*POLD1, POLE, MSH2*	Better	Mismatch repair deficiency (Rizvi et al., 2015)
*PTEN*	Worse	Increased immunosuppressive cytokines and attenuated T-cell infiltration and activity (Peng et al., 2016)
*EGFR*	Worse	Decreased PD-L1 expression and CD8 + TILs (Gainor et al., 2016; Haratani et al., 2017)
*JAK1, JAK2*	Worse	Insensitivity to IFNγ and its antiproliferative effects on cancer cells (Zaretsky et al., 2016)
*CALR, PDIA3, TAP1*	Worse	Impaired HLA-1 complex (Pereira et al., 2017)
*B2M*	Worse	Impaired MHC type I and HLA-1 molecules (Zaretsky et al., 2016; Janjigian et al., 2017; Pereira et al., 2017)
*PBRM1*	Better	Activation of JAK-STAT signaling pathway and elevated sensitivity to IFNγ (Miao et al., 2018; Pan et al., 2018)
*ARID2, BRD7*	Better	Enhanced sensitivity to T-cell-mediated cytotoxicity (Miao et al., 2018)
*MDM2/MDM4, DNMT3A*	Worse	Inhibition of p53 tumor suppressor (Kato et al., 2017)
*TERT, NF1, NOTCH1*	Better	Unclear (Kato et al., 2017)
*APLNR*	Worse	Attenuated IFNγ responses in tumors (Patel et al., 2017)


Relapse specific mutations were investigated and identified in four patients with required resistance to PD-1 blockade therapy in melanoma, including loss of function of *JAK1*, *JAK2*, and *B2M*, which induces either lack of response to interferon gamma (IFNγ), or loss of surface expression of major histocompatibility complex I (MHC I) (Zaretsky et al., 2016). Afterward, multiple clinical reports and subsequent experiments have confirmed that *B2M* alterations in tumor cells (i.e., mutations, deletions, and down-regulation) can largely induce acquired CPI resistance (Gettinger et al., 2017; Janjigian et al., 2017; Grasso et al., 2018). Importantly, high frequency of initial *B2M* mutations were found in patient-derived xenografts for lung cancer, suggesting patients with this gene mutation may experience primary resistance to CPIs (Pereira et al., 2017). With CRISPR screening, multiple genes were also identified to be essential for cancer immunotherapy, including *APLNR*, which can interact with *JAK1* (Patel et al., 2017). Therefore, alterations of these genes may also induce primary or acquired resistance. Clinically, it will be helpful to predict the poor responders and relapse risk by examining the alterations status of these resistance-related genes, which can be further considered as biomarkers.

Despite of point mutations, somatic copy number alterations (SCNAs) and structure variations (SVs) are also key hallmarks and driver events of tumorigenesis. Interestingly, most of the gene expression signatures exhibit down-regulation in high level of SCNAs tumors (also named aneuploidy tumors), including CD8 + T cell receptors and IFNγ pathways. Consistently, SCNA level is negatively related to the CPI treatment outcomes. Although paradoxically, SCNAs levels are positively correlated with the number of TMBs in 8 out of 12 tumor types, especially with passenger mutations. Combination of aneuploidy and TMB can increase the prediction efficiency to separate good and poor responders, indicating the potential of SCNAs as independent biomarkers (Davoli et al., 2017).

## Tumor Microenvironment

### Cells Contributing to Tumor Immunity

The TME includes not only tumor cells, but also extracellular matrix, stromal cells and immune cells, which closely interact with tumor itself. As the main force in anticancer immunity, the presence of TILs has been commonly considered as a favorable predictor for prognosis of cancers (Ruffini et al., 2009; Reissfelder et al., 2015; Brambilla et al., 2016). High baseline level of pre-existing CD8 + T cells as well as increase in tumor infiltrating CD8 + T cells during treatment has been found to be associated with better response of patients treated with anti-PD-1 therapy (Tumeh et al., 2014; Daud et al., 2016). In turn, anti-PD blockades also increase the number and restore the function of effector T cells during the treatment (Wei et al., 2017; Zhou et al., 2017). Interestingly, TMB and PD-L1 overexpression is correlated to presence of TILs (Herbst et al., 2014; Nishino et al., 2017). Also, DNA repair gene mutation is companied by prominent lymphocyte infiltrates, especially activated cytotoxic T cells.

Nonetheless, a recent study on gastric adenocarcinoma indicates that increasing CD8 + T cells are surprisingly correlated with impaired survival as well as higher PD-L1 expression, which marks an adaptive immune resistant microenvironment (Thompson et al., 2017). In some clinical studies, increased TIL density after the second dose of CPI instead of the baseline of TIL status was significantly associated with clinical CPI activities (Hamid et al., 2011; Tumeh et al., 2014). Moreover, an approach to systematically assessing intra- and peri-tumoral T cell infiltration, namely immunoscore, has been considered as a stronger predictor of prognosis as well as response to ICB therapies due to its integrated evaluation of immune features (Mlecnik et al., 2016; Voong et al., 2017). Both Tregs and myeloid derived suppressor cells (MDSCs) contribute to T cell dysfunction and TME immunosuppression, thus presenting profound impact on resistance to PD blockades (Kalathil et al., 2013). The comparison of anti-PD-1 sensitive and resistant patients reveals that Tregs partly preclude the efficacy of anti-PD-1 (Ngiow et al., 2015), and that depletion of Tregs can potentiate checkpoint inhibitors (Taylor et al., 2017). Nevertheless, it is reported that apoptotic Tregs sustain and even amplify their immunosuppressive function via the adenosine and A2A pathways under oxidative stress, which highlights oxidative pathway as a metabolic checkpoint controlling Tregs and thus affecting the effect of anti-PD (Maj et al., 2017). Moreover, it has been newly discovered that a canonical nuclear factor κB (NF-κB) subunit c-Rel plays an essential role in Treg function, and chemical inhibition of c-Rel impairs Treg-mediated immunosuppression and potentiates the effect of anti-PD-1 therapy (Grinberg-Bleyer et al., 2017). MDSCs proliferate during cancer, inflammation and infection, and perform the immunosuppressive function through restraining T-cell response. Reducing the number of MDSCs has been proved to be capable of enhancing antitumor effect of anti-PD-1 blockade (Orillion et al., 2017). Indoleamine-2, 3-dioxygenase (IDO) is a rate-limiting enzyme that controls tryptophan catabolism in tumor cells and MDSCs within the TME, which is recognized as an important microenvironmental factor that impairs cytotoxic T cell responses and survival (Schafer et al., 2016). The microsatellite instable subset of colorectal cancer, distinguished by high expression of IDO, poorly responds to anti-PD-1 therapy (Xiao and Freeman, 2015). On the contrary, IDO-knockout mice treated with anti-CTLA-4 or anti-PD-1/PD-L1 demonstrate significant tumor growth regression and prolonged survival, and combination treatment of IDO inhibitors and CTLA-4 blockade has achieved remarkable tumor rejection (Holmgaard et al., 2013). Importantly, combination of anti-PD-1 CPI and IDO inhibitor (e.g., epacadostat) can increase the objective response rate and prolong the overall survival in clinical trial phase I/II, however, surprisingly failed in phase III recently in 2018, with no benefit but increased ADR rate, possibly requiring a biomarker to distinguish the precious responders.

### Immunoregulatory Pathways Within TME

In addition to alterations in signaling pathways in tumor itself, a series of pathways within TME also regulate immune activities and thus impact on effect of anti-PD therapies. Epigenetic silencing of T helper 1 (TH1)-type chemokines, CXCL9 and CXCL10, precludes effector T cells from trafficking to the TME and directly interacting with tumor cells. And it has been proved that epigenetic modulators can restore T cell activities and increase T cell infiltration, thus strengthening the therapeutic efficacy of PD-L1 blockade (Peng et al., 2015). Moreover, the lack of response to PD-1 blockade has also been found related to a signature of TGFβ signaling, which renders T cell exclusion and blocked acquisition of TH1-effector phenotype. And inhibition of TGFβ signaling provokes antitumor activities and promotes tumor susceptibility to anti-PD therapies in colorectal cancer as well as urothelial cancer (Mariathasan et al., 2018; Tauriello et al., 2018). CD28/B7 costimulatory pathway is commonly known to be required for T cell proliferation and activation. It is newly discovered that PD-1/PD-L1 interaction suppresses T cell function primarily by CD28 inactivation, and the rescue of exhausted CD8 + T cells by PD blockades is strongly dependent on CD28 expression, which elucidates the important role of CD28/B7 costimulatory pathway as a response indicator for anti-PD therapies (Hui et al., 2017; Kamphorst et al., 2017). Interestingly, contrary to that elevated PD-L1 expression benefits the response to anti-PD therapy, upregulation of alternative immune checkpoints, notably T-cell immunoglobulin mucin-3 (TIM-3), is related to adaptive resistance. And subsequent addition of TIM-3 blocking antibody can significantly reverse the treatment failure of PD-1 blockade (Koyama et al., 2016).

Particularly, another important pathway is IFN signaling, including IFN type I and II. IFNγ, produced primarily by Th1 cells, NKT cells and NK cells (Farrar and Schreiber, 1993; Boehm et al., 1997), is abundantly generated and activated when ICBs enhance T cell responses (Liakou et al., 2008; Peng et al., 2012). As a pleiotropic and critical cytokine in host immune activities and tumor rejection (Dighe et al., 1994; Kaplan et al., 1998), IFNγ exerts its effects through a complex and orderly signaling pathway (Ikeda et al., 2002). Loss or deficiency of IFNγ signaling pathway may render disorders of host immune behavior and consequent insensitivity to immunotherapy (Kaplan et al., 1998; Dunn et al., 2005). In a study on metastatic melanoma described above, loss-of-function mutations in genes involved in IFNγ pathway (e.g., *JAK1* and *JAK2*) are found associated with relapse of patients who have shown initial response to anti-PD-1 therapy. And *in vitro* truncating mutations of *JAK1* and *JAK2* results in insensitivity to IFNγ and its antiproliferative effects on cancer cells (Zaretsky et al., 2016). IFNγ functions as an important inducer of PD-L1 on the surface of tumor cells (Taube et al., 2012), and patients who have a better response to PD-L1 blockade also have increased IFNγ and IFNγ-inducible chemokines (Herbst et al., 2014; Powles et al., 2014). These researches shed light on the vital role of defective IFNγ pathway in the clinical effect or prognosis of anti-PD therapies. Distinct from IFNγ, type I IFN within innate immune system is critical for T cell priming and tumor elimination through signaling on DCs and lack of type I IFN will result in limited useful T cells for reactivating of antitumor activities (Diamond et al., 2011; Fuertes et al., 2011). This is in consistence with the effect of type I IFN induced by radiotherapy (Lim et al., 2014). Moreover, radiation-induced type I IFN has been proved to increase expression of MHC class I and antigen recognition (Burnette et al., 2011; Deng et al., 2014b). Peritumoral injection of immunostimulatory RNA into immune-cell-poor melanomas has been observed to initiate a cytotoxic inflammatory response and tumor inhibition mediated by type I IFN. More importantly, the activation of type I IFN upregulates the expression of both PD-1 and PD-L1 and consequently leads to prolonged survival when PD-1 blockade is combined (Bald et al., 2014).

## Host-Related Factors

### Peripheral Blood Markers

Great interest has also been aroused in exploring biomarkers within serum or plasma due to the convenience of sample acquirement. In terms of immune cells, relatively high eosinophil count and lymphocyte count indicate favorable overall survival in patients with melanoma treated with Pembrolizumab (Weide et al., 2016). A pretreatment neutrophil-to-lymphocyte ratio (NLR) < 5 has been reported to be associated with improved survival of patients with NSCLC treated with Nivolumab (Bagley et al., 2017). The baseline frequency of CD14 + CD16-HLA-DRhi monocytes has also been found to strongly predict the response to anti-PD-1 of patients with melanoma (Krieg et al., 2018). Moreover, low lactate dehydrogenase (LDH) is related to the prognosis of patients receiving anti-PD-1 therapy. Studies on patients with melanoma reveal that patients with an elevated baseline LDH have a significantly shorter overall survival compared to patients with normal LDH, and the extent of increase in LDH during treatment is also correlated with the outcome of anti-PD-1 (Diem et al., 2016; Weide et al., 2016). Notably, a peripheral blood profiling reveals that clinical failure of anti-PD-1 therapy does not only result from insufficient host immune activation, but also depends on the ratio between circulating Ki-67-positive cytotoxic T cells and pretreatment tumor burden. Patients with higher ratio are more likely to exhibit improved response rate and survival (Huang et al., 2017), indicating that decreasing tumor burden by previously appropriate topical treatment may facilitate the effect of anti-PD therapy.

### MHC Class I and T-Cell Receptor (TCR)

MHC class I presenting antigen to cytotoxic T cells is an essential prerequisite for immune recognition and elimination of transformed cells (Aptsiauri et al., 2007). Downregulation of MHC class I has been acknowledged as a common mechanism of tumor immune escape and a potential determinant of clinical success of many immunotherapies (Haworth et al., 2015). Therefore, impaired MHC class I molecules have also been proposed as a candidate mechanism of resistance to anti-PD therapies, which has been reported to mainly result from deficiency in β2-microglobulin (B2M), a critical component of human MHC class I molecules (also named as HLA in human) required for CD8 + T cell recognition (Restifo et al., 1996; Wang et al., 2016; Zaretsky et al., 2016; Patel et al., 2017). Likewise, a study on lung cancer confirms that the loss of *B2M* is correlated with disrupted HLA-1 antigen processing and presentation, which leads to acquired resistance to PD-1 blockade (Gettinger et al., 2017). Another study also shows that factors which impair HLA-1 complex, including not only inactivation of *B2M* but also mutations at genes involved in maturation of HLA-1 complex (e.g., *CALR, PDIA3*, and *TAP1*), can affect the response to anti-PD-1/PD-L1 therapies (Pereira et al., 2017). In addition, the diversity of HLA-1 genotype also contributes to the outcome of anti-PD. It has been recently found that patients with maximal heterozygosity at HLA-I loci (*A, B*, and *C*) demonstrate improved overall survival compared to those who are homozygous for at least one HLA locus. Moreover, patients with HLA-B44 supertype have extended survival whereas HLA-B62 or somatic loss of heterozygosity in HLA-1 is related to poor outcome in melanoma cohorts (Chowell et al., 2017). Interestingly, loss of heterozygosity in HLA is revealed to be associated with a high neoantigen burden, upregulation of cytolytic activities and PD-L1 positivity, indicating the significance of combining multiple biomarkers to predict the response to PD-1/PD-L1 therapy (McGranahan et al., 2017).

Additionally, the variety of TCR repertoire is also related to clinical response. A more clonal and less diverse T cell repertoire is found in responding patients treated with anti-PD-1 (Tumeh et al., 2014), which is opposite to anti-CTLA-4 blockade (Postow et al., 2015b).

### Immune-Related Genetic Signatures

Mutations or altered expression of certain genes involved in host immune activities may reduce lymphocyte infiltration within tumors or compromise T cell functions (**Table 3**). As abovementioned, loss-of-function mutations in *B2M* gene lead to impaired MHC I molecules, and have been reported to be associated with acquired resistance to anti-PD therapies in melanoma, lung cancers and esophagogastric cancers (Zaretsky et al., 2016; Gettinger et al., 2017; Janjigian et al., 2017; Pereira et al., 2017). Particularly, in patients with *KRAS*-mutant lung adenocarcinoma, *STK11/LKB1* alterations are significantly associated with PD-L1 negativity and promote resistance to PD-1 inhibitors (Skoulidis et al., 2018). Furthermore, a study using a genome-scale CRISPR–Cas9 library profiles essential genes whose loss impairs the activity of CD8 + T cells, leading to resistance or non-responsiveness of cancer cells to T-cell-based immunotherapies. Notably, most of these genes play crucial roles in antigen presentation or IFNγ signaling (Patel et al., 2017). Interestingly, studies adopting the same approach newly discover that the loss-of-function mutations in *PBRM1*, which encodes a subunit of a SWI/SNF chromatin remodeling complex (the PBAF subtype), might improve the responsiveness to ICBs due to activation of JAK-STAT signaling pathway and elevated sensitivity to IFNγ in renal cell carcinoma (RCC) and melanoma, respectively. Apart from *PBRM1*, mutations of additional two genes which also encode components of the PBAF form of the SWI/SNF chromatin remodeling complex, *ARID2* and *BRD7*, are also found associated with the benefit of ICBs (Miao et al., 2018; Pan et al., 2018). Analysis of genomic alterations associated with accelerated tumor growth has found that *MDM2/MDM4* amplification is correlated with poor clinical outcome and even hyperprogression of patients after receiving anti-PD therapies. Besides, abnormalities of *EGFR* and *DNMT3A* also indicate a worse outcome, while alterations of *TERT, PTEN, NF1*, and *NOTCH1* appear to be related to better effect of anti-PD (Kato et al., 2017). A transcriptional signature, including up-expression of genes implicated in regulation of mesenchymal transition, cell adhesion, extracellular matrix remodeling, angiogenesis and wound healing, is indicated to be related to innate anti-PD-1 resistance (Hugo et al., 2016). Similarly, overexpression of genes involved with extracellular matrix (e.g., *LAMA3*) and neutrophil function (e.g., *CXCR2*) is related to progressing metastatic melanoma treated with PD-1 blockade (Ascierto et al., 2017). Changes in certain immune-related genes might lead to variations in the entire immune functions, hence genetically evaluation of the host immune status should be considered as a potential biomarker impacting on PD blockade immunotherapy.

## The Gut Microbiota

The intestinal microbiota contain a dominant part of innumerable bacteria in human bodies and are closely linked to host health through absorbing nutrients, degrading xenobiotics, regulating epithelial homeostasis and defending against potential pathogens (Eberl, 2010). Disorders in gut microbiota have been considered to participate in the development of not only colorectal cancer but also extraintestinal cancers (Brennan and Garrett, 2016; Loo et al., 2017). Previous studies have revealed the influence of gut microbiota on clinical efficacy of cancer chemotherapy (Iida et al., 2013; Viaud et al., 2013). Also, later investigations have found correlations between gut microbiome community and clinical response to ICBs. It is firstly found that effects of CTLA-4 blockade depend on distinct Bacteroides species, *B. thetaiotaomicron* or *B. fragilis* (Vetizou et al., 2015). Similarly, the anticancer immunity in mice models induced by anti-PD-L1 is reported to be associated with *Bifidobacterium*, which might improve effect through augmenting dendritic cell functions and subsequently enhancing CD8 + T cell priming and accumulation in the TME. And oral administration of *Bifidobacterium* alone generates equal effect on tumor eliminating as anti-PD-L1 does, indicating its potentially important role in strengthening immune functions (Sivan et al., 2015).

Recently, the predictive value of gut microbiota has been verified in human bodies. Routy et al. find that abnormal intestinal microbiota composition caused by antibiotics can lead to primary resistance to ICBs, and transplantation of fecal microbiota from patients who respond to ICBs into germ-free of non-responders can restore or enhance the responsiveness. Correlation has also been revealed between better clinical response to anti-PD-1 blockade and relative abundance of *Akkermansia muciniphila*, which increases the recruitment of CCR9 + CXCR3 + CD4 + T lymphocytes into tumor beds in a IL-12-dependant manner (Routy et al., 2017). A study on patients with melanoma unveils significantly different gut microbiota constitution between responders and non-responders treated with anti-PD-1 therapy. The gut microbiome of responding patients shows higher diversity and amplitude in *Ruminococcaceae bacteria*, while relatively less diverse bacteria and plenty of *Bacteroidales* are found in poorly responding patients. It is additionally found enrichment of anabolic pathways as well as enhanced systemic and anti-tumor immunity in responders (Gopalakrishnan et al., 2017). Similarly, another study on patients with melanoma also reveals a correlation between response to anti-PD-1 and abundance in more diversified bacteria, including *Bifidobacterium longum*, *Collinsella aerofaciens*, and *Enterococcus faecium* (Matson et al., 2018). Moreover, a study of the effect of pretreatment gut microbiota and metabolites on response in patients treated with different ICBs provides more diversified results. In terms of different regimens, the responders for all therapy types are enriched for *Bacteroides caccae*, the microbiota of the responders for Ipilimumab plus Nivolumab are rich in *Faecalibacterium prausnitzii*, *Bacteroides thetaiotaomicron*, and *Holdemania filiformis*, and that of the responders for Pembrolizumab contain abundant *Dorea formicogenerans*. High levels of anacardic acid are also found in ICB responders (Frankel et al., 2017). The findings above indicate that it is plausible to modulate gut microbiota to strengthen clinical effect of anti-PD therapy, yet more preclinical analyses of certain bacteria species and metabolites as well as confirmatory clinical studies are warranted. Moreover, gut microbiota is largely varied in terms of multiple factors, including ethnicity, living environment, diet habit, and etc, thus very difficult to guide the clinical practice as a biomarker.

## Combination Therapies With PD-1/PD-L1 Blockade

Hitherto, the remarkable outcomes of anti-PD therapies are merely observed in quite limited patients with certain types of cancers, while more patients fail to respond, exhibit resistance or relapse following treatment. Based on currently known mechanisms impacting clinical effect of anti-PD immunotherapy, combination therapies are required and being explored in order to improve response rate and expand benefited populations.

Adequate proliferation, smooth migration into tumors and complete function performing of effective T cells are fundamental requirements for the immune system to restrain tumor progression. Accordingly, epigenetic reprogramming drugs to facilitate T cell trafficking (Tan et al., 2007; McCabe et al., 2012), and targeting TNF family signaling pathways to strengthen T cell functions (Tolcher et al., 2017) have been developed and proved to be effective in combination with anti-PD therapy. In addition to positive roles of T cells which help combat tumor cells, the negative roles of immunosuppressive components which support tumor progression, including Tregs, MDSCs, some B7 family members, are unneglectable. Tregs express CTLA-4, which explains improved clinical success of combination of anti-CTLA-4 and anti-PD as abovementioned (Larkin et al., 2015; Hodi et al., 2016; Hellmann et al., 2017; Wolchok et al., 2017). Prostaglandin E2 (PGE2) and its key synthesizing enzyme cyclooxygenase 2 (COX2) can induce and recruit MDSCs in TME, and inhibition of COX2 has synergized anti-PD therapy in pre-clinical models (Li et al., 2016). Inhibitors targeting other immune checkpoints such as Tim-3, LAG3 and TIGIT have also been explored their synergetic effect aligned with PD therapy (Sakuishi et al., 2010; Li et al., 2012; Chauvin et al., 2015). PD-L1 expression is also a primary biomarker impacting on PD pathway blockade. Lately, it has been discovered that CDK4/6 inhibition elevate PD-L1 expression by restraining its degradation mediated by cyclin D-CDK4 and the SPOP ligase, and the combination of CDK4/6 inhibitors and anti-PD-1 therapy enhances tumor regression and dramatically improves overall survival of murine tumor models (Zhang J. et al., 2017). In terms of the field of vaccination, PD pathway blockade has been noticed to increase the antitumor effect of conventional vaccines, which can stimulate T cell activities and induce immune responses against tumor cells (Duraiswamy et al., 2013; Karyampudi et al., 2014). Another vaccination approach is oncolytic viral therapy. Locally injected oncolytic viruses have been proved to enhance systemic antitumor immunity through multiple mechanisms, thus improving the strength of anti-PD immunotherapy and elevating response rate of patients with advanced melanoma, brain tumors and breast cancer (Ribas et al., 2017; Bourgeois-Daigneault et al., 2018; Samson et al., 2018). Based on the significant role of metabolic fitness in immune activities, it has been reported that metformin, a broadly prescribed type II diabetes treatment, reverses the resistance to PD-1 blockade which results from hypoxic environments produced by tumors (Scharping et al., 2017). Conventional therapies targeting tumor cells, including radiotherapy and chemotherapy, also exert enhanced antitumor activities together with anti-PD therapy through multiple interacting mechanisms (Deng et al., 2014a; Shalapour et al., 2015; Sharabi et al., 2015; Twyman-Saint Victor et al., 2015; Shaverdian et al., 2017). However, more clinical evidence is needed to further determine appropriate doses, timing and other parameters in combination treatment. In addition, other potential combinatorial regimens have been considered and the confirmation trials are ongoing, such as tumor stromal fibroblast inhibitors and antibodies targeting innate immune signaling pathway and oncogenic signals (Mahoney et al., 2015; Sharma and Allison, 2015; Zou et al., 2016; **Table 4**).

**Table 4:** Effective therapeutic combinations with PD-1/PD-L1 blockade.

Target	Rationale	Combined therapy
T cells	Promoting effector T-cell trafficking into TME	Epigenetic reprogramming drugs
TNF family	Enforcing T-cell function	Utomilumab, a human IgG2 mAb agonist of the T-cell costimulatory receptor 4-1BB/CD137
Immunosuppressive networks	Depletion of Tregs	Anti-CTLA-4 antibody, ipilimumab
		Anti-CCR4 antibody, mogamulizumab
		CD73-specific antibody
	Inhibition of B7 family members (B7-H3, PD-L1)	B7-H3 blockade CDK4/6 inhibitors
	Blockade of other immune checkpoint inhibitors	Tim-3, LAG3 and TIGIT blockades
	Triggering innate immune system to achieve tumor destruction	Radiation therapy and chemotherapy
Cancer cells	Inhibiting oxygen consumption in tumor cells	Metformin
Tumor specific antigens	Increasing T cell infiltration	Oncolytic viral therapy
Inflammatory mediators	Decreasing MDSCs	COX2 inhibitors
Tumor stromal fibroblasts	Reducing CXCL12 produced by fibroblasts, which mediates immunosuppressive effect in pancreatic cancer.	CXCL12 receptor chemokine receptor 4 (CXCR4) inhibitor, AMD3100
	Blocking TGFβ signaling	TGFβ blockade
BRAF signaling pathway	Increasing the cross-presentation of antigens from dead tumor cells	BRAF inhibitors
MDSC-secreted factors	Inhibition of angiogenic factor VEGF	VEGF-specific antibody, bevacizumab
	Inhibition of cytokine receptor CSF1R, resulting in CD8 T cell infiltration into tumors	CSF1R inhibitors


## Conclusion

PD-1/PD-L1 pathway blockades have elicited outstanding clinical effect with relatively tolerable toxicities only in a minority of populations. In order to select patients most suitable to receive the possibly effective but costly therapy, the underlying prognostic factors leading to heterogeneous responses of different individuals with various cancer types have been gradually explored. In this review, a series of tumor-autonomous, tumor microenvironmental and host-related mechanisms were introduced, which need to be considered in terms of reducing ADR. With more and more prognostic factors gradually excavated, how to select most suitable biomarkers for certain cohorts is of great significance. Especially, the selection becomes more difficult when biomarkers predicting opposite response to anti-PD therapy present in one individual. For example, attenuated immune functions in elderly patients may result in poor clinical response of anti-PD with insufficient effector T cells, and on the other hand, the mutational burden accumulates with aging, which makes the outcome of anti-PD in elderly patients elusive. Unlike the traditional target therapy, which directly inhibit the abnormal signal in tumor itself (e.g., proliferation), CPI immunotherapy is more complicated and can be influenced by many factors. It has to be noted that some prognostic factors interact with each other instead of impacting the response of treatments independently. As aforementioned, virus infections and HLA heterozygosity are both associated with PD-L1 positivity or overexpression (Wong et al., 2015), while oppositely, genomic alterations are significantly related to PD-L1 negativity (Skoulidis et al., 2018). Loss of heterozygosity in HLA is additionally associated with a high neoantigen burden and upregulation of cytolytic activities (McGranahan et al., 2017). Besides, expression of the whole PD-1/PD-L axis, including PD-1, PD-L1, and PD-L2, has been reported to be connected with cytolytic activities and mutational load (Danilova et al., 2016). Above evidence indicates that it is necessary to exclude the impact of interactions between biomarkers and explore the independent roles of these candidates in larger patient cohorts with detailed information for all candidate biomarker, which will benefit the joint application of multiple biomarkers. Generally, sufficient infiltration and potent function of effector T cells in TME indicate an active pre-existing antitumor immunity and are the most elementary mechanism, through which most of other factors essentially impact on response of the therapy. Patients with abundant intratumoral infiltrate, elevated PD-L1 expression level and high mutational load have been most commonly reported to benefit from anti-PD therapies. Among all the influential factors, some were newly discovered and thus need to be verified and further explored, and some have been frequently reported but lack standard of measurement or practical application. Notably, there are contradictory findings in certain biomarkers. In terms of gut microbiota, some studies indicate a positive correlation between responses and *Bacteroides* species (Vetizou et al., 2015; Frankel et al., 2017), whereas the study of Gopalakrishnan et al. provides with an opposite finding that plenty of *Bacteroidales* are related to poor response to anti-PD-1 (Gopalakrishnan et al., 2017). The contradiction may be attributed to diversities in ethnics, region, diet, and limited sample sizes. Besides, the study of responding patients with RCC and NSCLC revealed different composition of beneficial gut microbiota from that of studies of melanoma (Routy et al., 2017), which emphasizes the role of different bacteria species in different cancer types, and indicates that all the biomarkers require validations in more cancer types. Based on currently known rationales, plenty of other therapies have been explored in combination with anti-PD therapies to improve benefit of previously poorly responsive populations. Although failed in some studies, precision designs with specific markers could provide insight on the combination therapy.

In conclusion, it is essential to comprehensively assess the patient’s status, especially with respect to the paradoxes, for instance, mutation loads and immunity in old patients and differences of beneficial bacteria in the above researches, etc. Besides, the differences in population and regions of patients should be taken into account. Finally, to adopt appropriate therapies, such as combination therapies, benefits the most for patients. Therefore, it is imperative to take comprehensive factors related to TME, host immunity, clinical factors and gut microbiome and so on into consideration when patients are given ICB therapies, which may shed new light on personalized precision therapy.

## Author Contributions

XY and SZ wrote the manuscript. YD and PW drew the figure. QH and HX contributed to the conception of the study.

## Conflict of Interest Statement

The authors declare that the research was conducted in the absence of any commercial or financial relationships that could be construed as a potential conflict of interest.
